# Well-distributed 1T/2H MoS_2_ nanocrystals in the N-doped nanoporous carbon framework by direct pyrolysis

**DOI:** 10.1038/s41598-023-34551-8

**Published:** 2023-05-09

**Authors:** Yalda Tarpoudi Baheri, Mahdi Maleki, Hossein Karimian, Jafar Javadpoor, Seyed Morteza Masoudpanah

**Affiliations:** 1 School of Metallurgy and Materials Engineering, Iran University of Science and Technology (IUST), Narmak, Tehran, 16846 Iran; 2 Department of Chemical Engineering, Golestan University, Aliabad Katoul, 45138-15739 Iran

**Keywords:** Energy science and technology, Materials science, Nanoscience and technology

## Abstract

Molybdenum disulfide (MoS_2_) has been a promising anode material in lithium-ion batteries (LIBs) because of its high theoretical capacity and large interlayer spacing. However, its intrinsic poor electrical conductivity and large volume changes during the lithiation/delithiation reactions limit its practical application. An efficient synthesis strategy was developed to prepare the MoS_2_ nanocrystals well-anchored into the N-doped nanoporous carbon framework to deal with these challenges by a confined reaction space in an acrylonitrile-based porous polymer during the carbonization process. The prepared hybrid material comprises small 1T/2H-MoS_2_ nanoparticles surrounded by a nanoporous carbon matrix. In addition to the highly crystalline nature of the synthesized MoS_2_, the low I_D_/I_G_ of the Raman spectrum demonstrated the development of graphitic domains in the carbon support during low-temperature pyrolysis (700 °C). This novel three-dimensional (3D) hierarchical composite shows superior advantages, such as decreased diffusion lengths of lithium ions, preventing the agglomeration of MoS_2_ nanocrystals, and maintaining the whole structural stability. The prepared C/MoS_2_ hybrid demonstrated fast rate performance and satisfactory cycling stability as an anode material for LIBs.

## Introduction

LIBs have become a primary power sources for portable electronic devices and electric vehicles due to their high energy/power densities, long life cycle, low self-discharge, and high operating voltage^1–3^. As one of the primary components, the anode material highly determines the LIBs performance. Carbonaceous materials, such as commercial graphite, graphene papers, carbon cloths, carbon nanotubes (CNTs), and carbon nanofibers, are widely utilized as LIBs anodes^4–7^. However, carbon-based anodes suffer from several drawbacks, such as low theoretical specific capacity, limited rate capability, and safety concerns, restricting LIBs development^8–11^.

Recent research works have been devoted to introducing novel anode materials with high capacity, reasonable cycle ability, and low cost^12–16^. Two-dimensional (2D) transition metal-sulfide anode materials with a high specific capacity, low cost, and other interesting properties have attracted considerable attention to be used as the LIBs anodes^17–19^. Among them, MoS_2_ is a promising candidate due to its unique layer structure, large interlayer spacing, weak interlayer van der Waals forces, adjustable band gap, the tailorable density of the active site, and high theoretical capacity (670 mAh g^−1^)^20,21^. Nonetheless, the practical application of MoS_2_ as a LIBs anode still suffers from drawbacks, including poor electronic/ionic conductivity, substantial volume expansion, and easy stacking/restacking causing fast capacity fading and rate performance deterioration during cycling^22–25^. The design of the nanostructure_,_ hybridization with conductive materials, and elemental doping have been practical solutions to improve the electrochemical performance of MoS_2_-based electrode materials^26–30^. Carbon-based frameworks can considerably enhance the ionic and electronic conductivity of MoS_2_^31–33^.

Hydrothermal (solution synthesis method) and chemical vapor deposition (CVD) procedures are the most frequent approaches for MoS_2_ deposition into carbonaceous frameworks as LIBs anodes^34^. The few-layer MoS_2_ vertically anchored on the nitrogen-doped graphene ribbons network has been synthesized through the hydrothermal method. Such composite 's convenient structure and high electrical conductivity led to the high electron/ion transfer kinetics and excellent rate capability of 499 mAh g^−1^ at a high current density of 8.0 A g^−1^^32,35^. Furthermore, the porous microspheres constructed from few-layered C/MoS_2_ nanosheets produced through a one-pot hydrothermal method exhibited a superior reversible capacity of 752 mAh g^−1^ at 0.2 A g^−1^^36^. The C/MoS_2_ hybrid has been also synthesized by a facile hydrothermal procedure using glucose additive as a carbon source in the presence of Mo and S precursors^37,38^. In the hydrothermal reaction environment, the glucose decomposition led a thin carbon layer formation around the small MoS_2_ nanosheets to reduce the diffusion path of lithium ions and speed up the reaction kinetics^37^. Although hydrothermal procedures are facile processes, it is difficult to deposit homogeneous coating of MoS_2_ with strong interfacial bonding and controlled thickness on the surface of the carbon networks^39^.

CVD methods have been utilized for in-situ grown-MoS_2_ layers on prefabricated carbon matrix^40^. The epitaxial growth of MoS_2_ nanohorns on the nanotubes (CNTs) network by the CVD method produced a composite with significant electrochemical performance and an excellent specific capacity of 982 mA h g^−1^ at 0.1 A g^−1^^41^. Graphenes were rolled up into hollow nanotubes, and fine MoS_2_ nanosheets were uniformly deposited on the interior surface of nanotubes to construct the MoS_2_@graphene nanocables using the CVD method. The prepared freestanding mechanically robust interwoven composite revealed a high specific capacity and excellent cycling performance^42^. However, CVD methods are complicated, costly, and time-consuming to coat the MoS_2_ on the carbon supports. Introduction of new deposition methods on the carbon framework without the challenges of the hydrothermal and CVD methods can develop the MoS_2_ application in the energy storage fields.

Notably, the morphology of the utilized carbon host and well-distributed MoS_2_ in the carbon matrix is insistent on volume change. The sufficient elastic space around the well-distributed MoS_2_ can reduce the electrode structure damage and active material detachment from the current collector. Furthermore, large three-interconnected pores can provide enough electrode–electrolyte contacting area^43^. Porous carbon structures such as graphene, CNTs, and amorphous carbons frameworks have been introduced as a matrix for MoS_2_^44–46^. However, most porous carbon-based hosts are poorly physically contacted with metal sulfides, leading to high charge-transfer resistance and even active materials detachment during cycling.

Therefore, developing well-dispersed MoS_2_ nanoparticles with strong interfacial bonding in hierarchical porous carbon frameworks is a straightforward way for obtaining high-performance LIBs anode. Herein, we report a novel method for well-distributed ultra-small MoS_2_ nanoparticles (less than 5 nm) in highly interconnected nanoporous carbon through a reaction between sulfur and molybdenum precursors in the confined spaces of a hierarchical polymeric polymerized high internal phase emulsion (polyHIPE). Carbonizing the infiltrated polyHIPE containing containing potassium persulfate (KPS) and sodium molybdate formed well-distributed and robust anchored small MoS_2_ nanoparticles in a carbonaceous bed. The high surface area C/MoS_2_ composite (242 m^2^ g^−1^) revealed high electron/ion transfer kinetics, good cycling stability, and rate capability.

## Experimental

### Materials

Sodium molybdate (Na_2_MoO_4_·2H_2_O), acrylonitrile (AN), divinylbenzene (DVB), 1,2- dichlorobenzene, benzoyl peroxide (BPO), potassium persulfate (K_2_S_2_O_8_, KPS), and calcium chloride dihydrate (CaCl_2_⋅2H_2_O) were purchased from Merck Co. (Darmstadt, Germany). Polyglycerol polyricinoleate (PGPR4150, Palsgaard, Denmark) was kindly given by PishgamanPakhsh Sedigh Co.

### PolyHIPE synthesis

AN as a monomer, DVB as a crosslinker, initiator (BPO), and the oil-soluble surfactant PGPR 4150 were added to DCB as the porogenic solvent. The aqueous phase was prepared by adding the CaCl_2_.2H_2_O and KPS to distilled water. Next, the aqueous phase was added dropwise into the organic phase to prepare the HIPEs. The emulsion was poured into a glass mold and heated at 70 °C for 24 h to obtain the polyHIPE. The polymerized emulsions were then dried at 60 °C for 24 h. The surfactant and unreacted monomers and initiators were extracted by ethanol and water for 24 h in a Soxhlet apparatus. The produced foam was stabilized under air at 240 °C for 8 h.

### C/MoS_2_ composite synthesis

**Figure 1:**
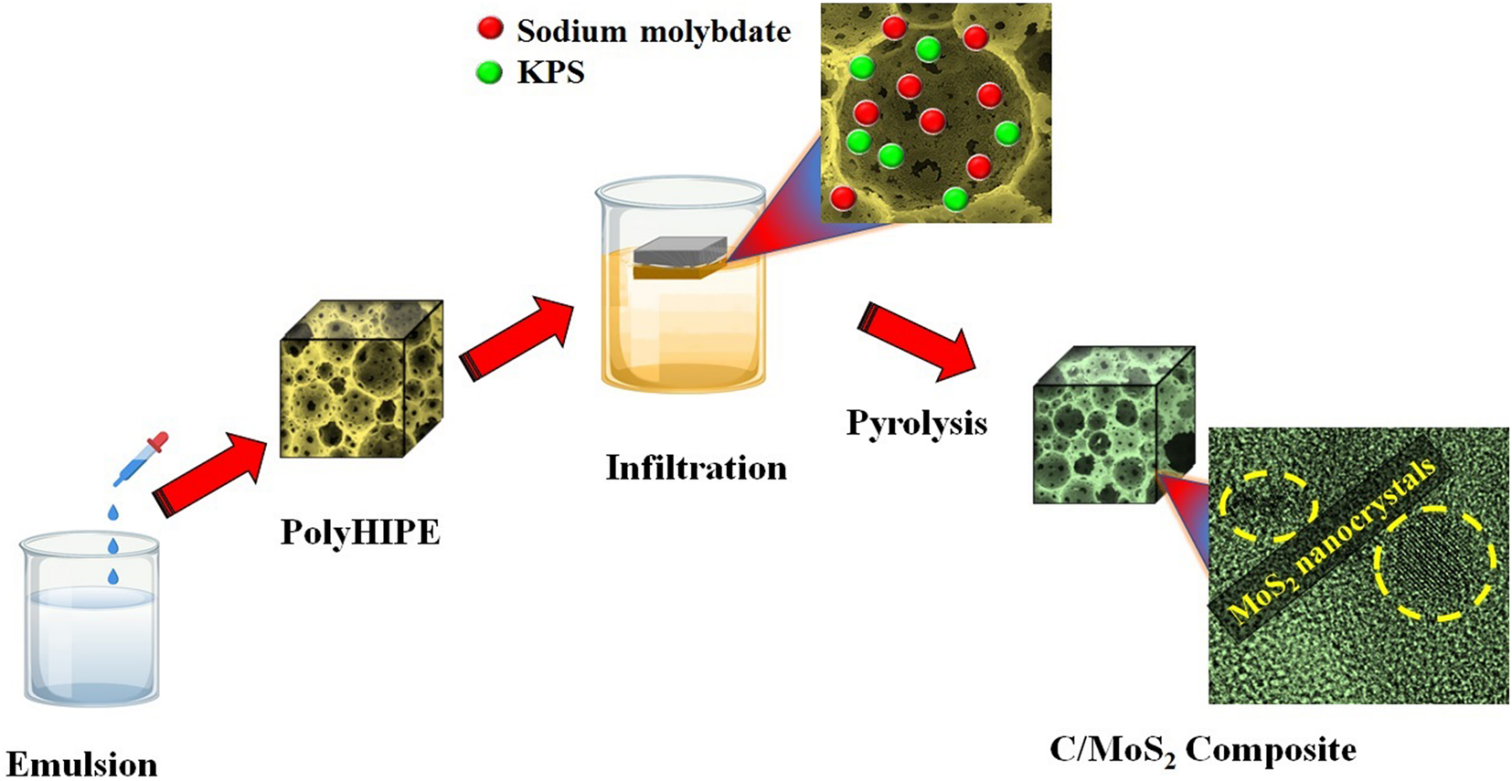
Scheme of the experimental route to obtain the well-distributed ultrasmall 1T/2H MoS_2_ nanoparticles in the carbon foam.

The prepared stabilized foam was infiltrated by ethanol/water (50/50 Wt%) solution containing sodium molybdate and KPS. Next, impregnated foams were pyrolyzed under a nitrogen atmosphere at 700 °C for 90 min. Figure 1 demonstrates a scheme of the experimental route to obtain the well-distributed ultrasmall 1T/2H MoS_2_ nanoparticles in the N-doped nanoporous carbon network.

## Characterization

An *X*'*Pert Pro MPD* diffractometer (X'Pert Pro MPD, Philips, Germany) was employed to record the X-ray diffraction (XRD) patterns. Nitrogen sorption analysis was measured using a Belsorb instrument at 77 K. To eliminate the adsorbed water and air pollutants, the composites were degassed under vacuum at 150 °C before analysis. X-ray photoelectron spectroscopy (XPS) was carried out with a KRATOS Axis Ultra system equipped with a monochromatized AlKα X-ray source, an aspherical mirror electron analyzer, and a charge neutralization system. SEM micrographs were captured by using a TESCAN VEGA// XMU microscope. A high-resolution transmission electron microscope (JEM-2100, JEOL, Japan) operating at 200 kV has been used for the microstructural investigations. The carbonaceous Au 4f. line (84.2 eV) was employed as the reference to calibrate the binding energies. Raman Spectroscopy was carried out on a Takram micro Raman spectrometer (Teksan™, Iran) equipped with a 532 nm laser as the excitation source at a power of 90 mW. Thermogravimetric analysis (TGA) was performed using a TA Instruments (SDT Q600, TA Instrument Co., USA ) at a heating rate of 10 °C min^-1^ in the air atmosphere.

## Electrochemical performance

The C/MoS_2_ composite, polyvinylidene difluoride (PVDF) binder, and conducting carbon (Super P) with a mass ratio of 8:1:1 were added to *N*-methyl-2-pyrrolidone (NMP) to obtain a homogeneous slurry. The slurry was then deposited uniformly on a copper foil. CR2032-type coin cells were assembled in an argon-filled glovebox with metal lithium foil as the reference and counter electrode and Celgard membrane as the separator. A 1.0 M LiPF_6_ in ethylene carbonate/dimethyl carbonate (EC/DMC, 1:1 volume ratio) was used as an electrolyte in the coin cell. Charge/discharge cycling was conducted on a computer-controlled Land CT2001A (Wuhan, China) battery tester at different current densities in a potential range of 0.01 − 3.0 V (vs. Li/Li^+^). The cyclic voltammetry (CV) measurement was conducted at a scan rate of 1 mV s^−1^ within the rage of 0.01–3.0 V on a CH Instrument 600D electrochemical workstation. Electrochemical impedance spectroscopy (EIS) was measured on an electrochemical work-station (VersaSTAT, Princeton Applied Research, USA).

## Results and discussion

### C/MoS_2_ formation

**Figure 2:**
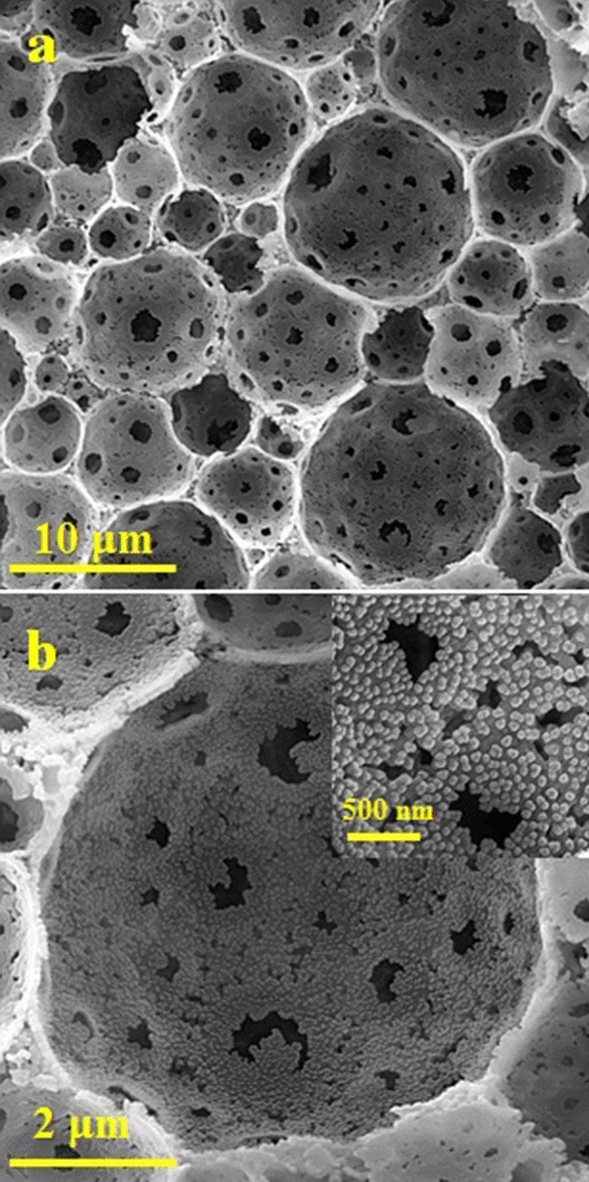
(**a,b**) SEM micrographs of the stabilized polyHIPE at different magnifications.

The prepared polyHIPEs were stabilized at 240ºC under an air atmosphere for 8 h to obtain the stable polymeric foam to accommodate the molybdenum and sulfur precursor. The SEM micrographs of the stabilized polymeric polyHIPE are shown in Fig. 2a,b. The distributed water droplets in the organic phase during emulsion polymerization led to 3D open cell interconnected porous structure. The measured average size of the cells and windows are 10 and 1 μm, respectively. The inner surface of the cells and windows demonstrates high roughness owing to intensive phase separation between AN and DVB as polymerizable precursors and DCB as the porogenic solvent.

**Figure 3:**
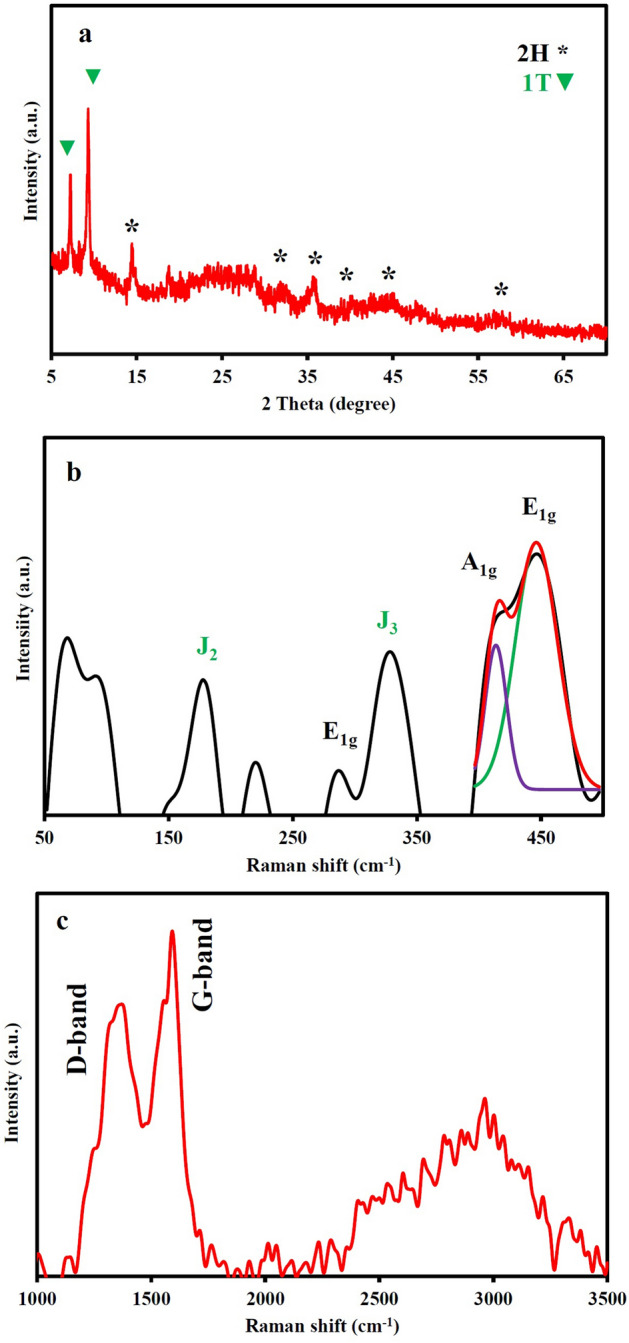
(**a**) XRD pattern and (**b,c**) Raman spectra of the prepared composite at 700 °C.

The pyrolysis of the infiltrated stabilized polyHIPE with Na_2_MoO_4_·2H_2_O and KPS led to a black bulk foamy structure. The crystal structure of the composite was investigated with XRD analysis (Fig. 3a). The diffraction peaks at 14.4, 32.5, 39.7, and 58° are respectively referred to the (002), (100), (103), and (110) crystallographic planes of the hexagonal MoS_2_ (2H phase, JCPDS No. 37-1492)^8^. In addition to 2H phase reflections, two sharp peaks are observed at low angles of 7.3° and 9.3°. It has been reported that the localization of carbon and nitrogen between MoS_2_ atomic layers during its formation led to the enlargement of MoS_2_ atomic interlayer spaces^47–50^. Owing to the presented synthesis procedure, Mo and S precursors can react in an interwoven hierarchical porous structure containing C, O, and N atoms. Therefore, the appearance of low-index peaks can be correlated to the interlayer expansion resulting from small intercalated atoms. Notably, in some research, the emerging peaks at 7.3° and 9.3° have been attributed to the formation of the 1T phase^51–53^. In addition to MoS_2_ peaks, the observed peak at 28.8° could be owing to the MoO_3_ hexagonal phase (JCPDS data card no. 21-0569).

Raman spectroscopy was performed to survey the MoS_2_ formation and graphitization development in the final composite (Fig. 3b). The broad peak in the range of 395–490 cm^−1^ is assigned to the 2H-MoS_2_ formation. The peak of 447 cm^−1^ is attributed to second-order Raman scattering 2LA(M) of MoS_2_^52,54^. The peaks at 219 (J_2_) and 329 cm^−1^ (J_3_) have been assigned to characteristic peaks of the 1T-MoS_2_ phonon mode^55^. The appearance of such peaks has been reported for the as-grown 1T MoS_2_ phase in the carbon fiber cloth matrix^56^. The peak appeared in the range of 90–100 cm^−1^ could be assigned to a vibrational feature in the molybdenum oxide compound detected in the exhibited XRD pattern^10^. The presence of an oxide compound has been detected in the exhibited XRD pattern. The Raman spectrum can demonstrate the graphitization degree development in the carbon matrix. As depicted in Fig. 3c, the Raman spectrum displays strong and distinct peaks at 1368 cm^−1^ and 1592 cm^−1^, arising from defective carbon bond (D-band) and sp^2^ hybridized carbon (G-band), respectively. The intensity ratio of the D and G bands (I_D_/I_G_), a common measure to evaluate the quality of graphitization degree in carbonaceous materials, was 0.8, suggesting a noticeable graphitization degree and high crystalline region density. The excellent I_D_/I_G_ ratio for the pyrolyzed sample at the low temperature of 700 °C is a notable finding compared to carbon-based materials synthesized at high temperatures^57,58^. Further, the broad peak between 2300 and 3250 cm^−1^ could be originated from developed graphitic domains in the microstructure.

**Figure 4:**
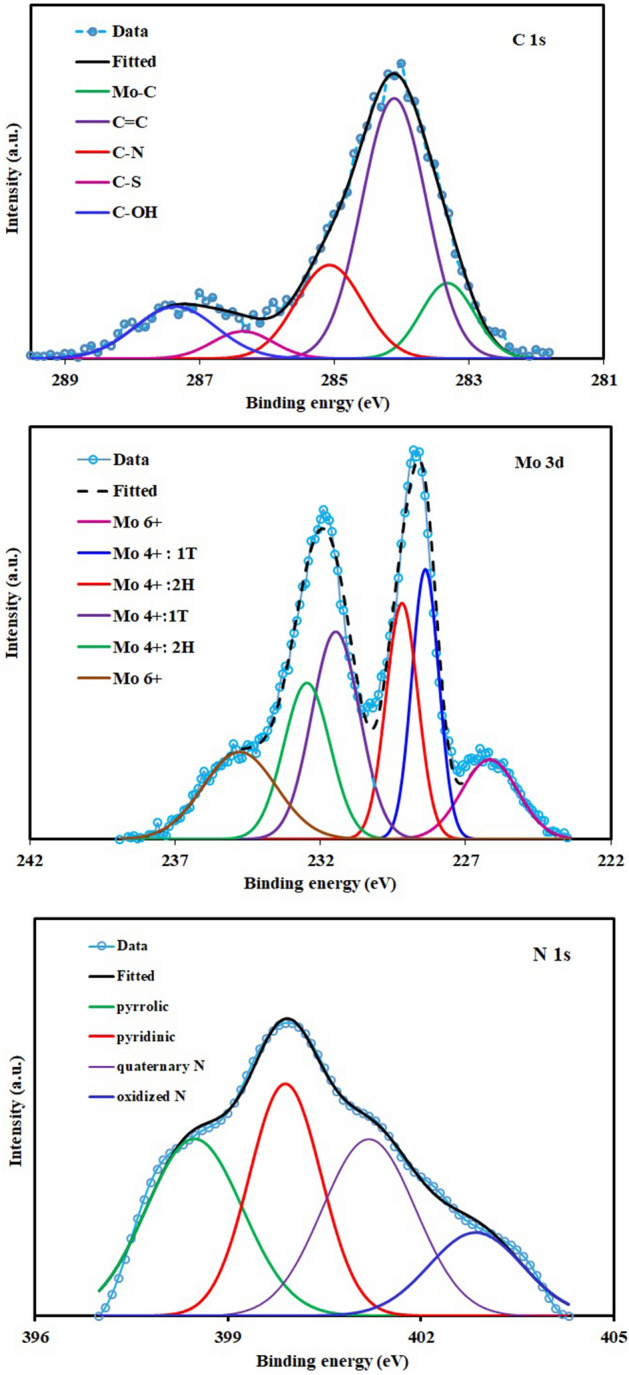
(**a**) C1s and (**b**) Mo 3d (**c**) N 1S XPS spectra of the prepared composite.

The successful formation of MoS_2_ in the carbon backbone was also investigated by XPS analysis (Fig. 4). The C 1s spectrum in Fig. 4a was deconvoluted into five peaks at 283.3, 284.1, 285.2, 286.3, and 287.3 eV using a Gaussian profile. The peak centered at 283.3 eV originates from the bonding between Mo and C atoms. The prominent peak with a binding energy of 284.1 eV is attributed to the C=C bonds. The peak at 285.2 eV can be attributed to C-N bonding in the carbon microstructure^59^. Due to the bonding between sulfur and carbon, a peak emerged at 286.3 eV. The peak at 287.3 eV is attributed to C–OH/C–O–Mo bonds or sp^3^ carbon in C–O^37,60^. The Mo 3d spectrum was deconvoluted into six peaks at 226.1, 228.4, 229.2, 231.4, 232.4 and 234.7 eV (Fig. 4b) using a Gaussian profile. The peaks at 228.4 eV and 231.4 eV could be, respectively, assigned to the 3d_5/2_ and 3d_3/2_ orbits of Mo^4+^ for 2H MoS_2_. The observed peaks at 229.2 eV and 232.4 eV are attributed to 3d_5/2_ and 3d_3/2_ of the Mo^4+^ for the 1T phase. The peaks appeared at 226.1 eV and 234.7 eV could be related to S 2 s and Mo^6+^, respectively^61–63^. The deconvolution of the N1s spectrum in Fig. 4c was fitted to four peaks including benzenoid amine (–NH–) or pyrrolic-type (399.4 eV), pyridinic (398.9 eV), quaternary N (401.2 eV), and oxidized N (402.8 eV)^64^. Acrylonitrile, well-known carbon precursor, has been utilized as a one of precursors to prepare the primary polymeric foam. This precursor has a nitrogen in its compound. When the pyrolysis temperature of polyacrylonitrile based polymer is lower than 900 °C, the nitrogen will present in the carbon microstructure^65,66^.

**Figure 5:**
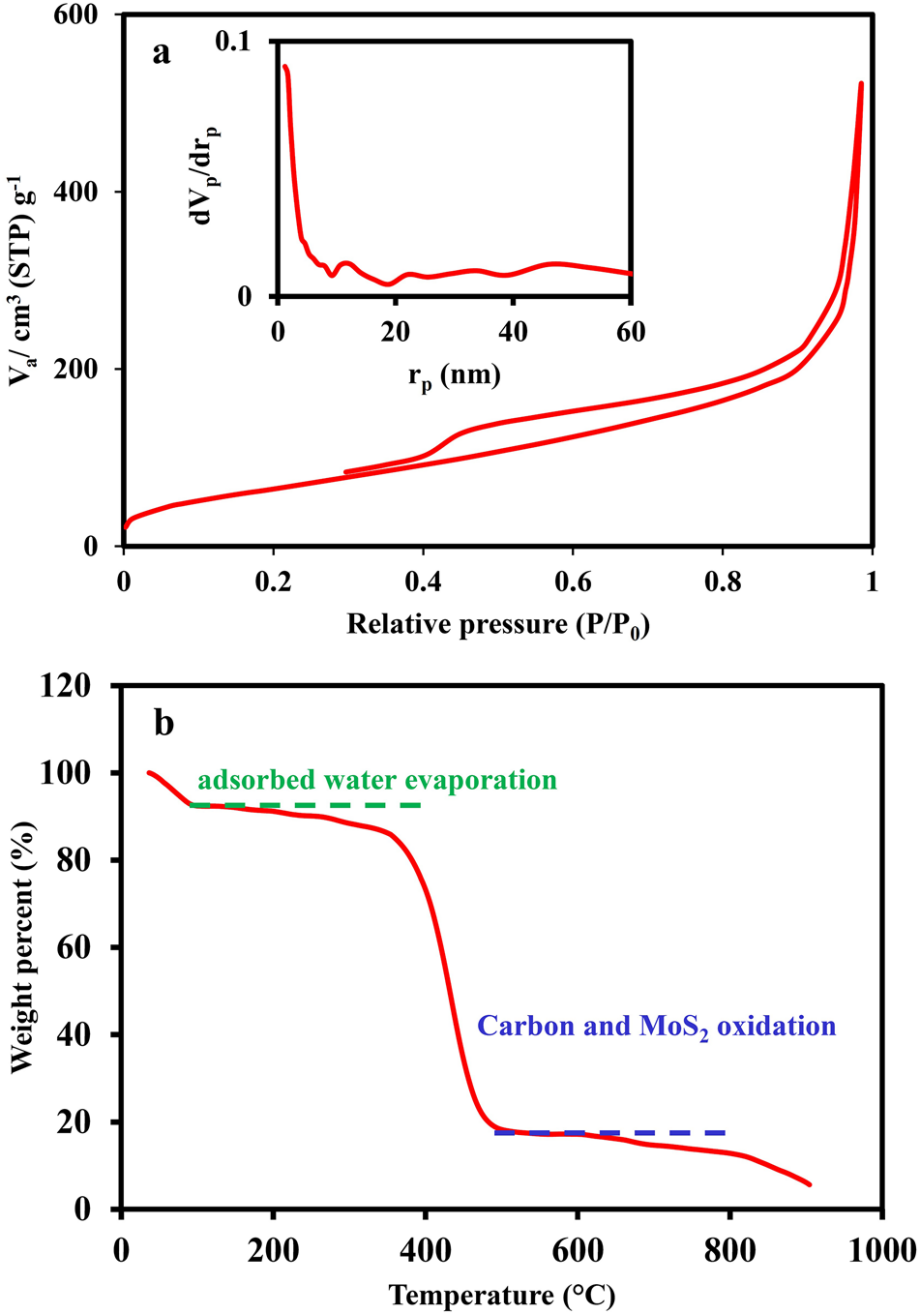
(**a**) N_2_ sorption isotherm and BJH diagram of the prepared C/MoS_2_ composite and BJH pore size distribution (inset) (**b**) TGA diagram of C/MoS_2_ composite under air atmosphere.

The N_2_ sorption isotherm of the C/MoS_2_ composite is depicted in Fig. 5a. According to International Union of Pure and Applied Chemistry (IUPAC) classification, the isotherm can be categorized as IV isotherm type and H3 hysteresis^3^. The presence of hysteresis demonstrates the formation of mesopores in the composite structure. The high gas uptake at high relative pressure can be related to big macropores. The BET surface area and pore volume of the C/MoS_2_ composite were 242 m^2^ g^−1^ and 0.8 cm^3^ g^−1^, respectively. The BJH pore size distribution of the composite in the inset of Fig. 5 demonstrates the hierarchical porous structure. The high surface area could be due to the highly porous carbon matrix and synthesis procedure. The MoS_2_ nanocrystals deposited on carbon-based supports by the hydrothermal method led to a surface area between 10 and 210 m^2^ g^−1^^67–69^. TGA analysis has been performed under the air atmosphere to determine the MoS_2_ content in the composite. As seen in Fig. 5b, weight loss was about 10%, when the temperature increased to 92 °C as a result of physically adsorbed water. The sharp weight loss appeared between 300 and 480 °C. Such weight reduction could be owing to the simultaneous oxidation of carbon to CO_2_ and MoS_2_ to MoO_3_^70–72^. The weight loss diagram shows a stable plateau between 530 and 640 °C which can be probably assigned to the remaining molybdenum oxide (17%). Therefore, the weight fraction of MoS_2_ in the composite is about 19%. The next weight reduction is related to the molybdenum oxide sublimation. Therefore, it can be deduced that the amount of MoS_2_ in the C/MoS_2_ composite can be about 15%.

**Figure 6:**
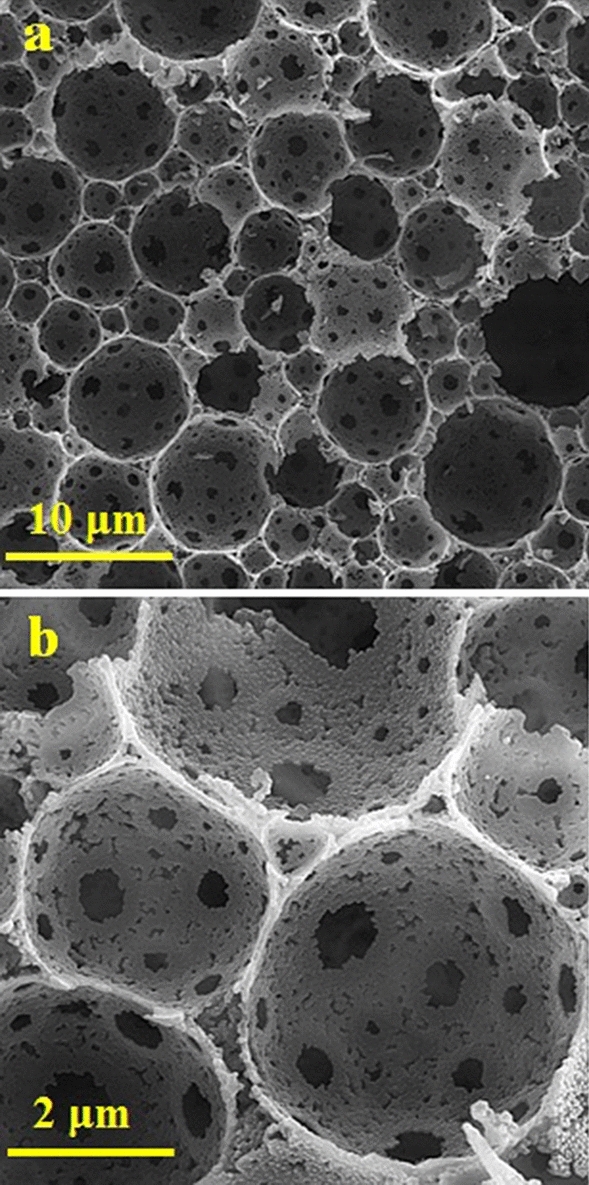
(**a,b**) SEM micrographs of the obtained C/MoS_2_ composite at 700 °C.

The SEM micrographs of the prepared composite are displayed in Fig. 6a, b. As shown, the carbon framework preserved the primary 3D-interconnected macroporous structure of polymeric foams during pyrolysis at 700 °C. Compared to former polymeric foam, the window and cell in the composite foam show 20 and 15% shrinkage, respectively. As can be seen, the surface of pore walls reveals a high roughness originating from the primary polymeric foam. The infiltration of MoS_2_ precursors in the initial polymeric foam shows no adverse effect on the porous carbon matrix structure.

**Figure 7:**
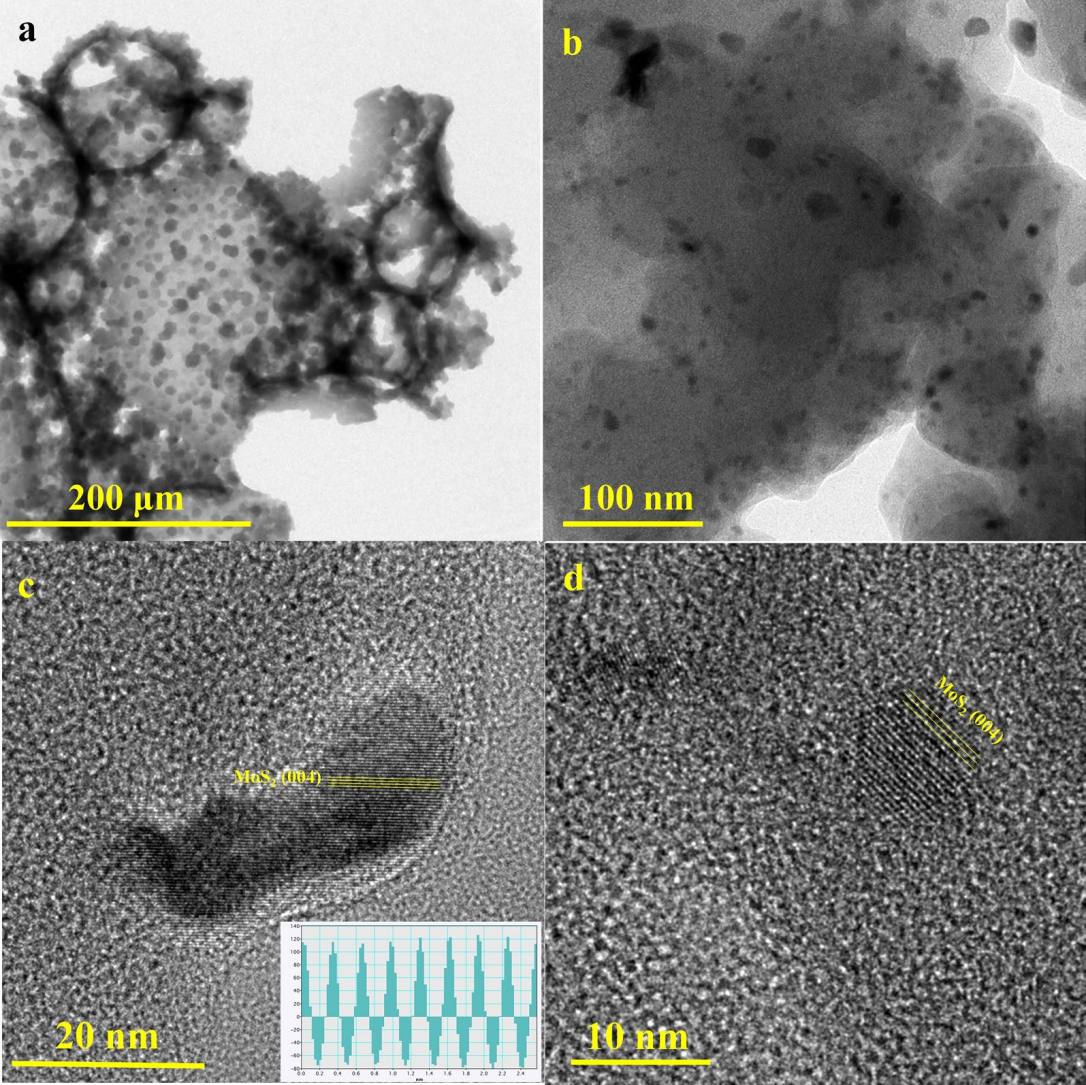
(**a**) TEM micrograph of the porous C/MoS_2_ composite (**b**) well-distribution of MoS_2_ distribution in carbon matrix, and (**c,d**) MoS_2_ nanoparticles crystallinity [inset of (**c**) shows d-spacing of MoS_2_ nanoparticles].

The TEM micrograph of the prepared composite in Fig. 7a displays a hierarchical porous structure confirming the BJH plot of the composite. The nanoporosity can be seen in the carbon microstructure. The well-distributed MoS_2_ nanoparticles (less than 10 nm) in the porous carbon matrix in Fig. 7b can be detected. The magnified images of the atomic-resolution image in Fig. 7c, d demonstrate the highly crystalline nature of the MoS_2_ nanoparticles. Inset of Fig. 7c displays d-spacing of 0.32 nm consistent with the (004) atomic planes of 2H-MoS_2_.

**Figure 8:**
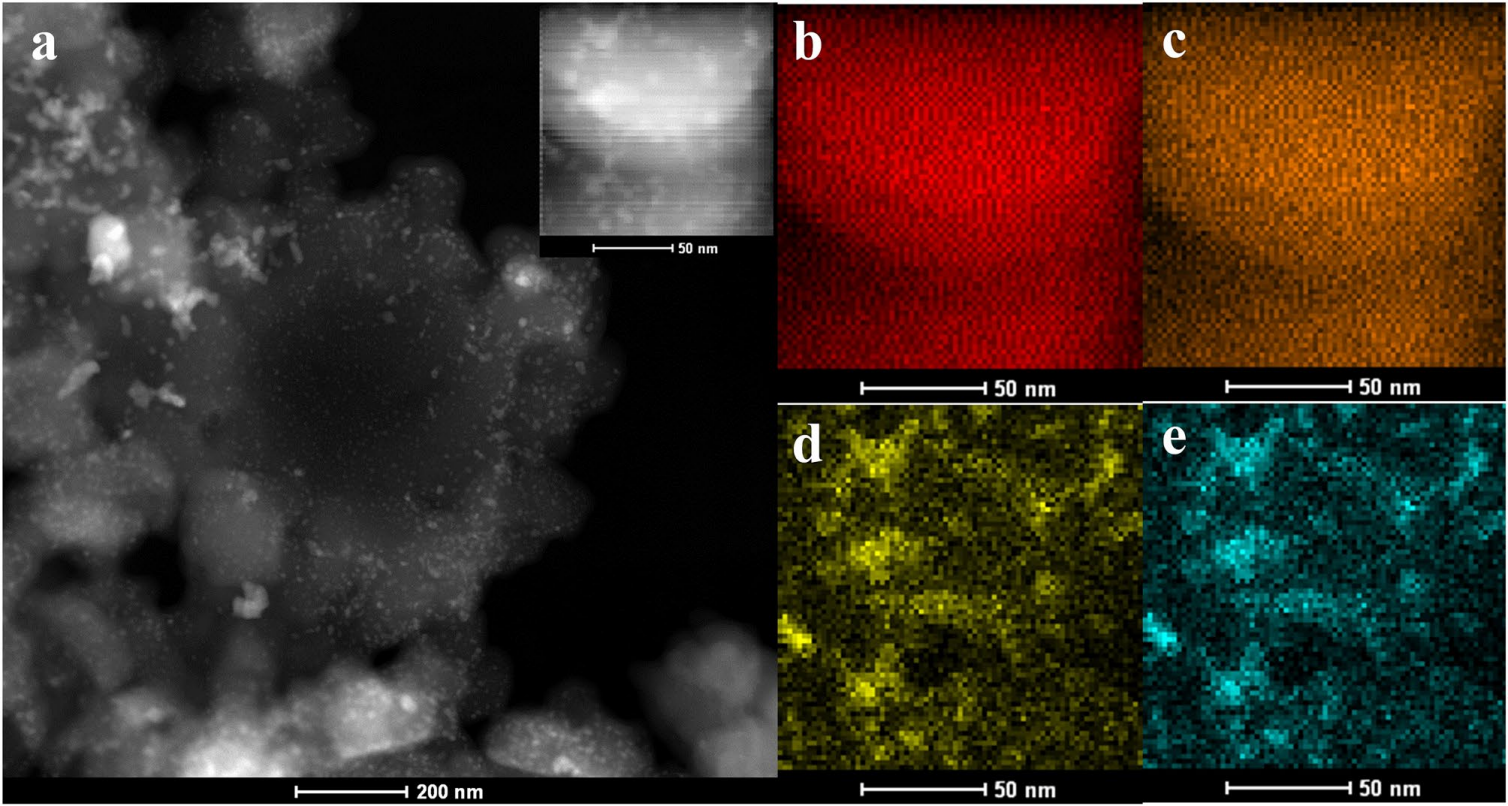
**(a)** HAADF-STEM and TEM image of C/MoS_2_ (inset) and (**b**) C, (**c**) N, (**d**) Mo, and (**e**) S elemental mapping images.

The HAADF-STEM image of the composite in Fig. 8 indicates the excellent dispersion of MoS_2_ nanoparticles in the carbon background. Figure 8 shows the EDS elemental maps for C, N, Mo, and S elements, indicating the distribution of the MoS_2_ nanoparticles in the carbon microstructure. Furthermore, the nitrogen map demonstrated the presence of this element in the composite microstructure which was confirmed before by XPS analysis.

### Electrochemical performance

**Figure 9:**
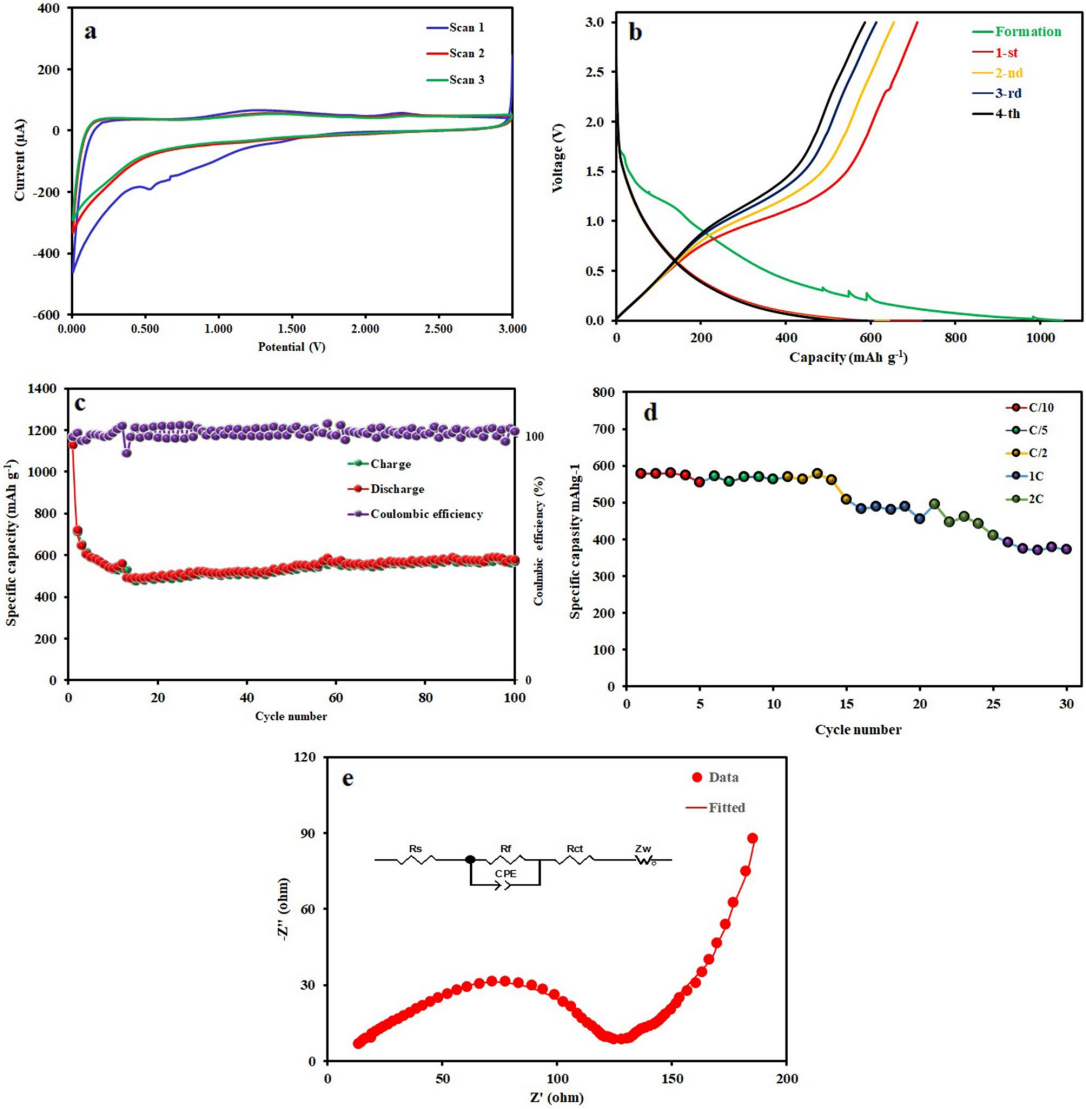
(**a**) The first three CV curves tested at a scan rate of 1 mV s^−1^, (**b**) discharge–charge voltage profiles from the 1st to 5th cycles with a current rate of 0.2 C in the potential window of 0.01–3.0 V (vs. Li/Li^+^), (**c**) cycle performance, and (**d**) rate capability, and (**e**) electrochemical impedance spectra and equivalent circuit model of the C/MoS_2_ composite.

The electrochemical performance of the C/MoS_2_ composite as anode materials for LIBs was assessed. Figure 9a shows the first three CV curves of the electrode at a scan rate of 1 mV s^-1^ within a potential range of 0.01–3.0 V. In the first cathodic sweep, three obvious reduction peaks at around 1.2–1.6, 0.66, and 0.53 V can be observed. The former corresponds to the insertion of Li^+^ into MoS_2_ interlayers to form Li_x_MoS_2_. The observed peaks at 0.66 and 0.53 V are attributed to the reduction of Mo^2+^ into the metallic Mo nanoparticles embedded into a Li_2_S matrix through the conversion process^37,73^. During the first anodic scan of the C/MoS_2_ electrode, two oxidative peaks at 1.2 V and 2.2 V are observed, which can be assigned to the oxidation of Li_2_S into S. In the anodic scanning, an oxidation peak appeared at approximately 2.25 V represents the oxidation reaction from Mo to Mo^4+^ and Mo^6+^. After the first cycle, the electrode is mainly composed of Mo and S instead of the initial MoS_2_. The anodic peaks at 1.4 and 2.3 V overlap with those in the second and third sweeps. During the anodic scan, the oxidation peaks at 1.4 and 2.3 V are the sulfide redox reaction which is related to the partial oxidation of Mo to MoS_2_ and the complete formation of MoS_2_^52,74^.

The electrochemical performance of the C/MoS_2_ composite was studied by galvanostatic charge–discharge tests. Figure 9b displays the discharge–charge voltage profiles of the C/MoS_2_ composite from the 1st to 5th cycles with a current rate of 0.2 C in the potential window of 0.01–3.0 V (vs. Li/Li^+^). The galvanostatic discharge curve of the C/MoS_2_ sample indicated an ultrahigh initial discharge capacity of 1128 mAh g^−1^. The primary discharge curve shows a short potential plateau between 1.25 and 1.1 V and a long plateau starting from ~ 1.1 V. The appearance of the short plateau is owing to the irreversible electrolyte decomposition and the solid-electrolyte interphase (SEI) film formation on the composite. Such SEI film formation leads to a fall in charge/discharge capacity^75^. As shown in Fig. 9c, the C/MoS_2_ composite electrode demonstrates a stable cycle performance at a current density of 0.2 C. The C/MoS_2_ composite preserved a capacity of 554 mAh g^−1^ across 100 cycles at 0.2 C with a high Columbic efficiency (~ 99.5%) (Fig. 9c). Figure 9d depicts the comparison of the rate capability of the prepared C/MoS_2_ composite. The C/MoS_2_ hybrid electrode displays an excellent rate capability of 565, 540, 500, 430, and 370 mAh g^–1^ at different current densities of 0.1, 0.2, 0.5, 1, 2, and 3 C, respectively. Even at a high current density of 3 C, the C/MoS_2_ composite electrode still delivers a high reversible capacity of about 370 mAh g^−1^. Therefore, the C/MoS_2_ composite anode can fast charge/discharge at various applied current densities.

The Nyquist plots in Fig. 9e exhibited a semicircle in the high- frequency region followed by a straight line in the low frequency region. The intercept in the high-frequency region corresponds to the ohmic resistance of the cell (R_e_), which combines the total resistance of the electrolyte, separator, and electrical contacts. The diameter of the semicircle is representative of charge transfer resistance (R_ct_). As can be seen, the R_e_ value was very low (13 Ω) and R_ct_ was 120 Ω which indicates the quick charge transfer process of the lithium-ion insertion/extraction reaction. The straight line in low frequency representing the Warburg resistance (Z_W_) is assigned to the solid-state diffusion resistance of Li ions in the electrode, respectively^17^.

The uniform distributed molybdenum and sulfur precursors in initial polyHIPE led to the uniform growth of MoS_2_ nanoparticles in the carbon matrix. Further, the hierarchical porous structure of the carbon matrix can provide appropriate channels for electrolyte penetration and ion transportation. The well-dispersed ultrasmall MoS_2_ nanoparticles led to the extended electrode/electrolyte interface with highly porous carbon that facilitates ion diffusion into the inner structure of the electrode materials. Despite the low amount of MoS_2_ in the composite based on TGA result, MoS_2_ pinning on the carbon matrix and strong interactions between the MoS_2_ and carbon components prevent the MoS_2_ restacking and the stability enhancement of the C/MoS_2_. Furthermore, the carbon template prevented the overgrowth and aggregation of MoS_2_ into bulk materials during the cycling process.

## Conclusion

Herein a novel strategy was developed to grow the 1T/2H-MoS_2_ nanocrystals in the N-doped nanoporous carbon. The pyrolysis of sodium molybdate and KPS in the acrylonitrile-based polymer backbone led to the formation of the pinned ultrasmall MoS_2_ nanoparticle in the highly porous carbon matrix. The high surface area of the hybrid structure led to a short lithium diffusion path and easier lithiation/delithiation in the microstructure. The C/MoS_2_ composite delivered a high initial capacity of 556 mAh g^−1^ at 0.2 A g^−1^, and its capacity was maintained even after 100 cycles, showing high cycling stability.

## Data Availability

The datasets used and/or analyzed during the current study available from the corresponding author on reasonable request.
